# Cell-Based Biosensors in Oral Health: Emerging Tools for Rapid Detection and Monitoring of Oral Diseases

**DOI:** 10.3390/bios16050254

**Published:** 2026-04-30

**Authors:** Florinel Cosmin Bida, Ionut Luchian, Dana Gabriela Budala, Dragos Ioan Virvescu, Costin Iulian Lupu, Oana Maria Butnaru, Teona Tudorici, Florin Razvan Curca, Ovidiu Aungurencei, Andrei Georgescu

**Affiliations:** Grigore T. Popa University of Medicine and Pharmacy, 700115 Iasi, Romania

**Keywords:** cell-based biosensors, oral health diagnostics, salivary diagnostics, periodontal biomarkers

## Abstract

Oral diseases remain highly prevalent worldwide and require early diagnosis and continuous monitoring to improve clinical outcomes. Conventional diagnostic methods are often invasive, time-consuming, and limited in their capacity for real-time assessment, which has driven the development of biosensor technologies for point-of-care applications. Among these, cell-based biosensors utilize living cells as sensing elements capable of responding to inflammatory mediators, bacterial toxins, metabolic products, and tumor-associated biomarkers. This narrative review summarizes the principles, cell types, detection mechanisms, and applications of cell-based biosensors in oral health. The literature was identified through a structured search of PubMed, Scopus, Web of Science, and Google Scholar using keywords related to cell-based biosensors, oral diagnostics, salivary biomarkers, periodontal disease, oral cancer, and lab-on-chip technologies. Due to the heterogeneity of biosensor designs and detection methods, the selected studies were analyzed qualitatively. Cell-based biosensors have demonstrated applications in periodontal disease detection, cariogenic biofilm monitoring, oral cancer diagnostics, cytotoxicity testing of dental materials, and salivary biomarker analysis. The integration of microfluidic and lab-on-chip systems enables real-time and multiplex detection, supporting the development of chairside diagnostic platforms in dentistry. However, challenges related to standardization, reproducibility, and clinical validation remain and must be addressed to facilitate broader implementation in routine practice

## 1. Introduction

Oral diseases remain among the most prevalent non-communicable conditions worldwide, affecting billions of individuals and imposing a substantial clinical and socioeconomic burden [1]. Early detection and continuous monitoring are essential for improving therapeutic outcomes and minimizing long-term complications. However, conventional diagnostic approaches in dentistry—such as clinical examination, radiographic assessment, histopathological analysis, and laboratory-based biomarker testing—are often limited by the lack of real-time capability, the need for invasive sampling, and reliance on centralized laboratory infrastructure [2].

In recent years, biosensor technologies have emerged as promising tools for rapid, point-of-care diagnostics [3]. Among these, cell-based biosensors represent a novel and biologically responsive approach. Unlike conventional chemical or antibody-based sensors, these systems employ living cells, either engineered or naturally responsive, to detect specific molecular stimuli [4]. Cell-based biosensors are capable of identifying inflammatory mediators, bacterial products, metabolic alterations, and tumor-associated biomarkers by converting biological responses into measurable electrical, optical, or biochemical signals.

In the context of oral health, cell-based biosensors present significant potential due to the accessibility of biological fluids such as saliva and gingival crevicular fluid as diagnostic media [5]. In particular, saliva-based diagnostics provide a non-invasive platform for monitoring a wide range of biomarkers, including inflammatory cytokines, matrix metalloproteinases, microbial metabolites, and cancer-associated markers [6].

Despite recent technological advances, several challenges still limit the widespread clinical implementation of cell-based biosensors in dentistry. These include issues related to device miniaturization, cell stability, reproducibility, integration into point-of-care diagnostic platforms, and regulatory approval pathways [7]. Furthermore, the successful translation of experimental biosensor systems into clinically applicable diagnostic tools requires multidisciplinary collaboration across biotechnology, dentistry, materials science, and bioengineering [8].

The literature on cell-based biosensors in oral health is dispersed across dentistry, biomedical, and bioengineering research domains, despite the extensive investigation of biosensor technologies and salivary diagnostics. While numerous studies address either biosensor engineering concepts or salivary biomarkers independently, only a limited number explore the integrated application of cell-based sensing technologies with microfluidic systems, the evaluation of biocompatibility of dental materials, and their use in oral disease diagnostics.

Therefore, this review aims to provide an integrated overview of cell-based biosensor principles, cell models, detection mechanisms, and their specific applications in oral health, including periodontal disease, caries detection, oral cancer diagnostics, cytotoxicity testing, and salivary biomarker monitoring.

## 2. Materials and Methods

There is extensive usage of biosensors in biotechnology, environmental monitoring, and medical diagnostics; these analytical instruments transform a biological response into a quantifiable signal. Three primary parts make up a standard biosensor: a transducer, a signal processing system, and a biological recognition element. While the transducer transforms the biological interaction into a measurable electrical, optical, thermal, or electrochemical signal, the biological recognition element interacts with the target analyte [9].

Enzymes, antibodies, nucleic acids, aptamers, and molecularly imprinted polymers (MIPs) are typical recognition elements used in conventional biosensors [10,11,12]. Use of these biosensors for biomarker analysis, pathogen detection, and glucose monitoring has become widespread. Salivary indicators linked to periodontal disease and dental caries, including cytokines, matrix metalloproteinases, and bacterial metabolites, can be detected by biosensors developed for use in dentistry [13]. When it comes to complicated biological reactions like inflammation, cellular stress, or tissue disintegration, however, conventional biosensors often only pick up on individual molecules [14].

Nanotechnology, microfluidics, and microelectronics have made substantial progress in the last several years toward miniaturization, real-time detection, and enhanced sensitivity of biosensors. The rise in point-of-care diagnostic devices has been made possible by these technical breakthroughs. These devices can be employed in clinical settings without the requirement for complex laboratory infrastructure [15].

✓ Literature Search

The literature included in this review was identified through a structured search of major scientific databases, including PubMed, Scopus, Web of Science, and Google Scholar. The search strategy focused on studies related to biosensors, cell-based biosensors, oral diagnostics, salivary diagnostics, cytotoxicity testing of dental materials, microfluidic systems, and lab-on-chip technologies in oral health. The search was performed using combinations of the following keywords: “cell-based biosensors”, “oral biosensors”, “salivary diagnostics”, “periodontal biomarkers”, “oral cancer biomarkers”, “cytotoxicity testing dental materials”, “impedance biosensors”, “microfluidics oral diagnostics”, and “lab-on-chip dentistry”. Boolean operators (AND, OR) were used to refine the search and identify relevant publications. The search included studies published up to December 2025.

✓ Study Selection Criteria

Articles published in English in peer-reviewed journals were considered. Both experimental studies and review articles were included in order to provide a comprehensive overview of current developments in cell-based biosensors applied to oral health. Studies focusing on biosensor technologies in biomedical fields with potential applications in dentistry were also included.

The inclusion criteria consisted of studies describing cell-based biosensor principles, detection mechanisms, applications in oral disease diagnostics, salivary biomarker monitoring, cytotoxicity testing of dental materials, and microfluidic or lab-on-chip biosensor systems. Studies not related to biosensor technologies, oral diagnostics, or cell-based sensing mechanisms were excluded.

✓ Study selection Process

The initial database search yielded a total of 312 records across all databases. After removal of duplicate entries (*n* = 78), 234 records remained for screening. Titles and abstracts were screened, leading to the exclusion of 162 studies that were not relevant to the scope of the review (e.g., not related to oral health, not involving biosensor technologies, or not addressing cell-based sensing mechanisms).

The remaining 72 articles underwent full-text evaluation. In addition, 30 relevant studies were identified through manual cross-referencing of reference lists and were included following eligibility assessment.

This process resulted in a total of 102 references included in the present review. Among these, 8 references were used to support the introductory section, while 94 studies formed the basis of the qualitative synthesis and thematic analysis.

Given the narrative nature of this review and the heterogeneity of biosensor designs, cell models, and detection methods, a formal PRISMA flow diagram was not constructed. However, a structured and stepwise selection process was applied to ensure inclusion of the most relevant and recent studies.

✓ Data Extraction and Analysis

The selected studies were analyzed qualitatively, focusing on biosensor type, cell type used, detection mechanism, target biomarkers, analytical performance, and potential clinical applications in oral health. Due to the heterogeneity of biosensor designs, cell models, and detection methods, a quantitative meta-analysis was not feasible. Therefore, the results were synthesized narratively, highlighting current trends, technological developments, and clinical relevance of cell-based biosensors in oral diagnostics and monitoring.

## 3. Literature Review

### 3.1. Cell-Based Biosensors: Principles and Mechanisms

A subset of biosensors known as cell-based biosensors employs actual living cells to detect environmental changes. Receptor activation, gene expression, metabolic changes, or changes in cell morphology and viability are some of the physiologically relevant processes that cell-based biosensors respond to, in contrast to traditional biosensors that depend on isolated biomolecules [16].

Toxins, inflammatory mediators, bacterial virulence factors, yeast cells, or modified cell lines tailored to react to particular stimuli like tumor-associated biomarkers, bacterial virulence factors, or mammalian cells are the usual ingredients in these biosensors [17]. Variations in electrical impedance, luminescence, metabolic activity, oxygen consumption, and fluorescence intensity are among the observable reactions that cells produce when exposed to a target analyte [18].

**Figure 1:**
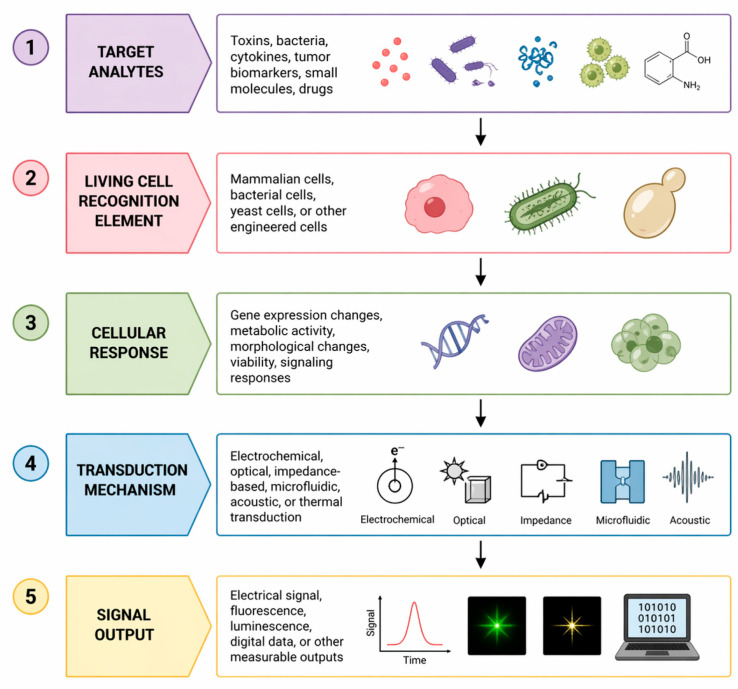
Working principle of cell-based biosensors: target analytes interact with living cells, inducing cellular responses that are detected by transducers and converted into measurable signals.

Cells are capable of responding to a broad spectrum of stimuli, including toxins, bacterial virulence factors, inflammatory mediators, tumor-associated biomarkers, and pharmacological agents such as drugs [19]. These stimuli induce cellular responses including changes in gene expression, metabolism, morphology, and cell viability. The cellular responses are then converted into measurable signals through various types of transducers, including electrochemical, optical, impedance-based, and microfluidic systems [20]. The general principle of cell-based biosensors and the signal transduction process is illustrated in Figure 1.

Cell-based biosensors can be classified into several categories based on detection mechanisms, including electrochemical cell-based biosensors, optical cell-based biosensors, impedance-based biosensors, and microfluidic lab-on-a-chip systems [21]. Impedance-based cell-based biosensors are particularly important in biomedical applications because they allow real-time monitoring of cell behavior, including cell adhesion, proliferation, cytotoxicity, and inflammatory responses [22].

Compared to traditional biosensors, which can only detect a single molecular marker, cell-based biosensors can detect multiple markers simultaneously, which is an enormous advantage [23]. For example, toxicity, tumor cell activity, infection, inflammation, and other complicated biological processes can be detected in this way. Regulatory clearance for clinical use, device stability, repeatability, and cell viability are some of the issues that cell-based biosensors offer [24].

This capacity of cell-based biosensors to detect toxicity, inflammation, infection, or tumor activity—even in situations where the specific biomarker is unclear or if many pathways are at work—is a significant benefit. Since there are several host-microbe interactions and inflammatory pathways involved in complicated multifactorial diseases like periodontitis, oral cancer, and bio-film-mediated disorders, cell-based biosensors are ideal for these types of conditions.

### 3.2. Cell Types Used in Cell-Based Biosensors

Different types of cells have been used in cell-based biosensors depending on the target analyte and the intended application. Bacterial cells are often used for detecting toxins, environmental pollutants, and microbial metabolites due to their rapid response and ease of genetic modification [25]. Yeast cells are also used in biosensing applications because they are easy to culture and can be genetically engineered to produce measurable signals in response to specific stimuli [26].

Cells derived from mammals are utilized extensively in biological biosensors because of their ability to detect inflammatory mediators, growth factors, toxins, and tumor-associated biomarkers. These cells are particularly valuable because they can mimic physiological cellular responses and signaling pathways that occur in human tissues, making them highly relevant for biomedical and oral health applications [27]. Mammalian cells used in biosensors include epithelial cells, fibroblasts, osteoblast-like cells, periodontal ligament cells, and various cancer cell lines, depending on the intended application [28].

Mammalian cells undergo changes in gene expression, metabolic activity, cell shape, proliferation, or apoptosis in response to certain analytes like bacterial toxins, oxidative stress factors, inflammatory cytokines, or tumor-related biomarkers. Then, detecting methods based on impedance, optics, or electrochemistry can measure these biological reactions [28].

Research on oral health often makes use of specific cell types, such as those from the gingiva, periodontal ligament, epithelium, osteoblasts, and oral cancer cell lines [29]. Periodontal pathogen-induced inflammatory responses, cytotoxic dental material impacts, and tumor-related indicators can all be detected by these cells [30].

**Figure 2:**
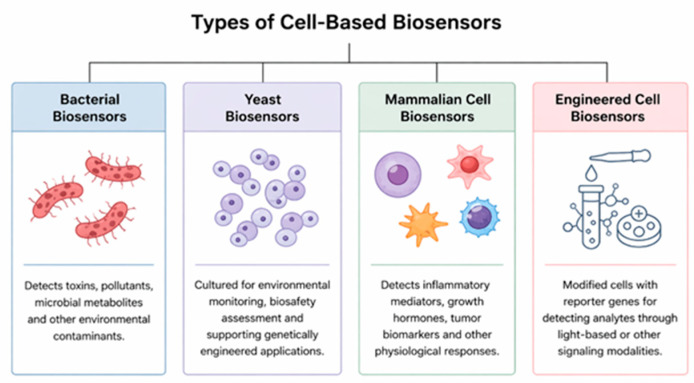
Cell types employed in cell-based biosensor technologies.

Additionally, cells that have been genetically modified can have reporter genes that emit light when certain signaling pathways, including those for inflammatory cytokines or oxidative stress, are engaged [31]. A growing number of biosensors are based on reporter cell systems, which enable the detection of biological reactions with a high degree of sensitivity. The main cell types used in cell-based biosensor technologies are summarized in Figure 2.

In the reviewed literature, commonly used cell models include both primary cells and established cell lines relevant to oral tissues and disease models. These comprise primary human gingival epithelial cells (HGECs) and human gingival fibroblasts (HGFs), as well as widely used immortalized cell lines such as MG-63 and SaOS-2 for osteoblast-like responses, THP-1 for immune-related assays, and oral squamous cell carcinoma cell lines (e.g., SCC-9, SCC-25, CAL 27) for cancer-related biosensing applications.

These models are frequently employed due to their reproducibility, availability, and relevance for specific biological responses in biosensor-based detection systems. However, detailed experimental parameters such as passage number, culture conditions, and cell handling protocols vary considerably across studies and are not consistently reported in the literature.

### 3.3. Detection Mechanisms and Signal Transduction

The detection mechanisms employed in cell-based biosensors depend on both the type of transducer used and the nature of the biological response being measured [32]. Electrochemical biosensors detect variations in electrical parameters such as current, voltage, or impedance resulting from cellular activity. Among these, impedance-based cell-based biosensors are widely applied for real-time monitoring of cell adhesion, proliferation, cytotoxicity, and inflammatory responses [32].

Optical biosensors enable the detection of cellular responses to specific stimuli by monitoring changes in absorbance, luminescence, or fluorescence generated by living cells [33]. These systems are particularly advantageous in cell-based applications, as optical signals can be measured non-invasively and in real time without compromising cell viability [33]. Fluorescent reporter gene systems are commonly employed to assess gene expression changes associated with inflammation, oxidative stress, cytotoxicity, or tumor cell activity [34]. In such systems, reporter genes encoding fluorescent proteins, such as green fluorescent protein (GFP), are activated in response to specific signaling pathways, enabling the detection of cellular responses at the molecular level [34].

Luminescence-based biosensors are widely employed to assess metabolic activity, ATP production, and the activation of specific cellular signaling pathways [35]. These systems typically rely on luciferase-based reporter assays, in which light emission is proportional to cellular metabolic activity or gene expression levels. Consequently, luminescent biosensors are extensively used in applications such as toxicity testing, drug screening, and cancer research [35].

Optical biosensors provide several advantages, including high sensitivity, real-time monitoring, non-destructive analysis, and the capability for multiplex detection of multiple cellular responses simultaneously [33]. However, these systems often require specialized instrumentation, such as fluorescence microscopes or spectrophotometers, and may be affected by signal interference arising from background fluorescence or light scattering. Despite these limitations, optical cell-based biosensors remain among the most widely utilized technologies in biomedical and oral health research [33].

To facilitate comparison between different biosensor approaches, a structured overview of major cell-based biosensor technologies, including their detection principles, applications, advantages, and limitations, is provided in Table 1.

Electrochemical biosensors are among the most widely used detection techniques in cell-based biosensing due to their high sensitivity, rapid response time, and compatibility with miniaturized devices [36]. These systems monitor changes in electrical parameters such as impedance, conductivity, voltage, or current, which are generated by cellular activity. When analytes such as toxins, inflammatory mediators, or bacterial metabolites interact with living cells, they can induce alterations in cellular metabolism and membrane properties, resulting in measurable electrochemical signals [36].

Electrochemical cell-based biosensors have been widely applied in oral diagnostics for the detection of salivary biomarkers associated with periodontal disease, including inflammatory mediators such as interleukins, tumor necrosis factor, and matrix metalloproteinases [37]. These systems are also capable of identifying metabolic by-products generated by cariogenic bacteria and periodontal pathogens. In addition, electrochemical biosensors have been investigated for the detection of oral cancer-related biomarkers in saliva, including specific proteins, nucleic acids, and metabolic indicators [38].

A major advantage of electrochemical biosensors lies in their compatibility with portable devices and point-of-care diagnostic platforms, making them particularly suitable for chairside applications in dental practice [39].

Among electrochemical approaches, impedance-based biosensors represent a widely used strategy for monitoring cytotoxic effects, cell adhesion, proliferation, and morphological changes. These systems typically incorporate microelectrode arrays, enabling the detection and recording of impedance variations associated with cellular processes such as adhesion, migration, proliferation, and cell death [40].

To determine the cytotoxicity of dental materials such as resin composites, adhesives, ceramics, and implant materials, researchers in the field of oral health frequently employ impedance-based cell-based biosensors [41]. For the purpose of studying implant osseointegration, these methods are also employed to examine cell adherence and proliferation on implant surfaces [42]. In addition, biosensors based on impedance can be employed to track the inflammatory reactions of cells in the periodontal ligament or the gingival fibroblasts when they are exposed to bacterial toxins or inflammatory mediators linked to periodontal disease [43].

Among the most encouraging advancements in cell-based biosensor technology, microfluidic and lab-on-a-chip technologies stand out, especially when it comes to oral health diagnostics. Systems like this use miniature platforms with integrated microchannels, sensors, and detection systems to analyze samples as tiny as saliva or gingival crevicular fluid [44].

**Figure 3:**
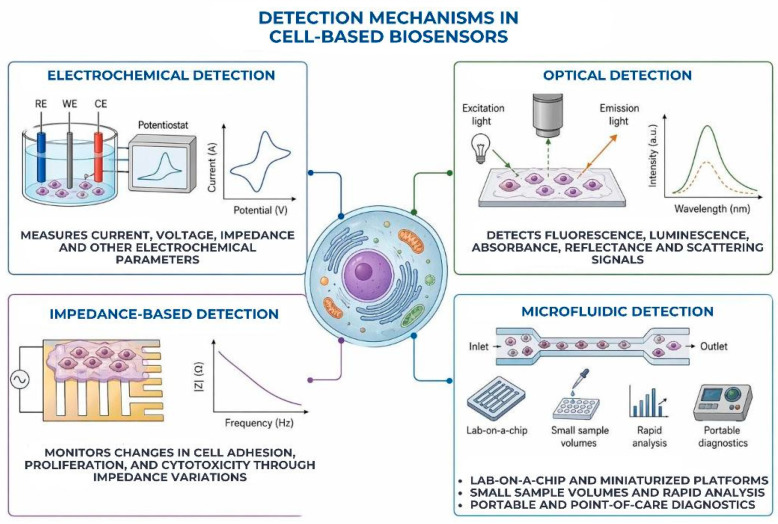
Detection mechanisms in cell-based biosensors, including electrochemical, optical, and impedance-based approaches, as well as microfluidic and lab-on-chip platforms for real-time analysis.

Oral cancer biomarker detection, periodontal disease monitoring, microbiological detection, and salivary diagnostics are all possible with microfluidic cell-based biosensors. These setups make it possible to track cellular reactions to inflammatory mediators, bacterial toxins, or salivary biomarkers in real time [45]. In order to study disease mechanisms and test potential therapeutic drugs, lab-on-a-chip devices can also be utilized to create models of periodontal tissue, biofilm, and oral mucosa [46]. The main detection mechanisms used in cell-based biosensors are presented in Figure 3.

### 3.4. Integration with Microfluidics and Lab-on-Chip Systems

A highly encouraging path for the future of periodontal diagnostics is the combination of salivary diagnostics with microfluidic and lab-on-chip platforms [47]. While traditional laboratory tests can yield valuable insights, they are frequently hindered by factors such as extensive processing periods, the requirement for specialized personnel, the sheer volume of samples needed, and their limited applicability to everyday dental procedures. The quick, sensitive, and possibly multiplex detection of salivary biomarkers in a portable and tiny format is made possible by microfluidic devices, which allow the manipulation of minute fluid volumes within microscale channels [48]. Since a quick and non-invasive evaluation of periodontal health is becoming more and more desirable, these features make lab-on-chip platform systems ideal for usage at the chairside [49].

**Figure 4:**
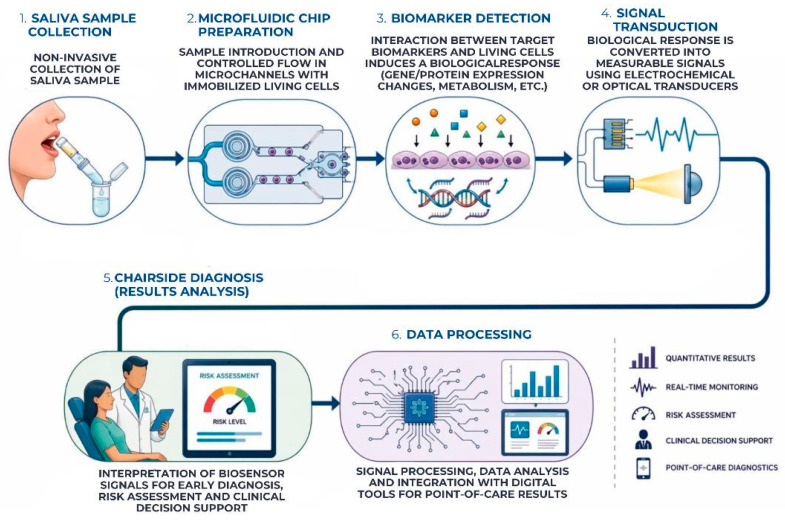
Microfluidic and lab-on-chip platform for salivary biomarker detection in periodontal diagnostics, enabling integrated sample processing, signal transduction, and chairside diagnosis.

Because it is non-invasive, easy to collect, and abundant in host-derived, microbial, and inflammatory indicators relevant to periodontitis, microfluidic devices are particularly beneficial to salivary diagnostics [50]. These systems can detect biomarkers like interleukins, matrix metalloproteinases, enzymes, oxidative stress mediators, and microbial DNA with minimal sample volumes by integrating immunoassays, nucleic acid detection modules, and electrochemical or optical sensing components. The development of genuine point-of-care periodontal testing is supported by their ability to execute numerous analytical processes on a single miniaturized platform [51]. These phases include sample preparation, separation, reaction, and signal readout. The workflow of microfluidic and lab-on-chip systems used for salivary biomarker detection in periodontal diagnostics is illustrated in Figure 4.

The ability to multiplex, or measure many biomarkers at once, is an immense advantage of lab-on-chip technology over traditional methods that rely on a single analyte [52]. The intricate interplay between dysbiotic biofilms and host inflammatory responses drives periodontitis, a multifactorial illness. This is of special relevance in this context. The beginning, activity, progression, and response to treatment can all be captured by different salivary markers [53].

Thus, a more reliable diagnostic profile could be achieved by integrating platforms that incorporate inflammatory mediators like IL-1β, IL-6, TNF-α, and MMP-8 with microbial signatures or genetic material. Multiplex biosensing and microfluidic line-of-sight devices are being developed at a rapid pace to overcome this restriction of traditional single-marker methods, according to recent reviews [54].

Another important strength of these systems is their compatibility with chairside and decentralized testing. Oral fluid-based point-of-care technologies are increasingly valued because they are affordable, simple to use, and rapid, making them highly relevant for busy dental settings [55]. In periodontal care, this could allow clinicians not only to support diagnosis at the first visit, but also to monitor treatment response, detect persistent inflammation, and stratify patients according to individual risk. Such applications align closely with the broader concept of precision dentistry, in which clinical decisions are supported by molecular and biochemical profiles rather than by retrospective clinical findings alone [55].

Microfluidic systems are becoming more and more significant as experimental platforms for periodontal biology modelling, in addition to their importance in diagnostic detection. Dynamic periodontal environment aspects, including fluid flow, shear stress, host-microbe interactions, and barrier function, can be reproduced by organ-on-chip and micro physiological systems, which are challenging to accomplish with static in vitro models [56]. Oral biofilm behavior, reactions of gingival and periodontal tissues, and interactions with biomaterials are being studied using these platforms in a more realistic setting.

Despite their promise, several challenges continue to limit the widespread clinical translation of microfluidic and lab-on-chip technologies in periodontology. These include issues related to device standardization, reproducibility, calibration, sample variability, biofouling, cost-effectiveness, and the need for validation in large and diverse patient cohorts. In addition, saliva itself is a complex biological matrix whose composition may be influenced by circadian rhythm, hydration, medication, smoking, systemic disease, and collection methodology [57].

As a result, the performance of miniaturized diagnostic platforms depends not only on sensor sensitivity but also on robust pre-analytical control and biomarker standardization. Regulatory approval, integration into dental workflows, and clinician training will also be essential before these technologies can become routine in daily periodontal practice [58]. The main challenges and limitations associated with microfluidic and lab-on-chip technologies in periodontal diagnostics are summarized in Table 2.

### 3.5. Applications for Cell-Based Biosensors in Oral Health

Cell-based biosensors have emerged as innovative tools for the detection and monitoring of periodontal disease by enabling real-time assessment of host–microbe interactions, inflammatory responses, and tissue responses to bacterial toxins and metabolic products. Unlike conventional diagnostic methods that rely primarily on clinical parameters such as probing depth, clinical attachment loss, and radiographic bone loss, cell-based biosensors allow the detection of biological changes at the cellular level before clinical signs become evident.

#### 3.5.1. Periodontal Disease

Gingival epithelial cells, fibroblasts, osteoblasts, or immune cells are frequently used as biological sensing elements in cell-based biosensors in periodontal research. In reaction to periodontal infections including *Porphyromonas gingivalis*, *Aggregatibacter actinomycetemcomitans*, and *Fusobacterium nucleatum*, these cells release cytokines, reactive oxygen species, and enzymes that split apart the matrix [68]. To indirectly provide biologically relevant information regarding pathogenic activity, biosensors can assess these cellular responses by electrical impedance, visual signals, metabolic activity, or reporter gene expression [69].

Rather than only detecting the existence of bacteria, one significant application is detecting their virulence. While conventional microbiological testing can detect bacterial contamination, it cannot tell whether the germs are actively damaging tissues [70]. On the other hand, biosensors based on cells can identify disease-related processes more precisely, such as cytotoxic effects, inflammatory responses, and breakdown of the barrier. Because of this, they are more effective at detecting current periodontal disease than past or chronic forms of the condition [71].

Furthermore, cell-based biosensors can be used to evaluate patient-specific inflammatory responses. Periodontitis progression depends not only on bacterial load but also on the host immune response [72]. By exposing cultured gingival or immune cells to patient saliva or plaque samples and measuring cellular responses, it may be possible to assess individual susceptibility, disease activity, and treatment response [73].

Such approaches support the concept of personalized periodontal diagnostics and precision dentistry. The main cellular models, detected biomarkers, detection methods, and clinical applications are summarized in Table 3.

#### 3.5.2. Dental Caries and Microbial Detection

Cell-based biosensors also play an important role in the detection of cariogenic microorganisms and in the study of microbial metabolic activity associated with dental caries [78]. Dental caries is a biofilm-mediated disease characterized by acid production from carbohydrate metabolism, leading to enamel and dentin demineralization. Traditional caries detection methods focus on visual examination, radiography, or microbial culture, but these approaches do not always reflect real-time bacterial activity [79].

For studies on cavities, biosensors can make use of epithelial cells, bacterial reporter strains, or synthetic cells that react to acidity, bacterial toxins, or certain compounds produced by microbes like lactic acid. Biosensor cells may undergo electrochemical, optical, or impedance-based alterations in respiration, metabolic stress, or inflammatory signaling when exposed to cariogenic bacteria like *Streptococcus mutans* or *Lactobacillus* species [80].

The identification of metabolic activity, as opposed to just the existence, of bacteria is another significant application. Acid generation, extracellular polysaccharide synthesis, and biofilm growth are all directly implicated in caries progression; several biosensors are designed to detect these processes [81]. Caries risk is dependent on bacterial activity and biofilm behavior, not merely bacterial numbers. This method gives more clinically relevant information than basic bacterial counts.

**Figure 5:**
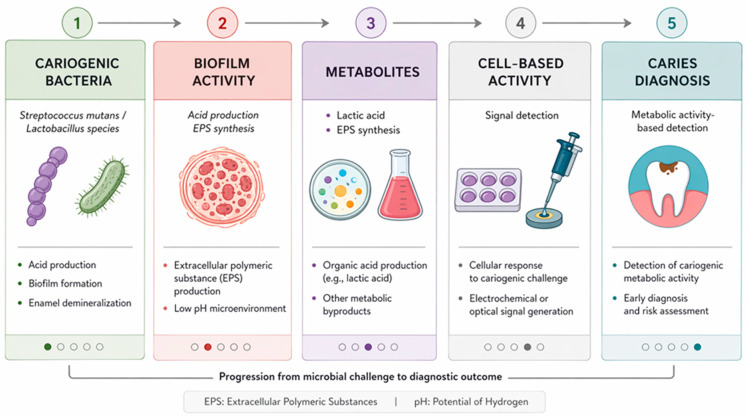
Principle of cell-based biosensors for dental caries detection, based on sensing bacterial metabolic activity and biofilm formation and converting these responses into measurable signals.

Incorporating cell-based biosensors into microfluidic systems allows for the investigation of biofilm growth in real-time, simulating saliva flow and nutrition supply [82]. Fluoride, antimicrobials, probiotics, and nutritional interventions can all be tested in controlled settings using these systems to see how well they work against cariogenic biofilms. The principle of cell-based biosensors for detecting cariogenic activity and biofilm metabolism is illustrated in Figure 5.

#### 3.5.3. Oral Cancer Detection

One of the most promising applications of cell-based biosensors in oral health is the early detection of oral cancer and potentially malignant disorders. Early diagnosis of oral squamous cell carcinoma significantly improves prognosis, but current diagnostic methods often rely on visual examination followed by biopsy, which may delay diagnosis [83].

Saliva and other oral fluids can be tested for carcinogenic substances, oxidative stress, DNA damage, and tumor-related indicators using cell-based biosensors. By activating particular signaling pathways, inducing apoptosis, changing metabolism, or expressing reporter proteins like fluorescent or luminescent markers, these systems use genetically engineered reporter cells, epithelial cells, or mammalian cells as biological sensors that react to carcinogenic or toxic substances [84].

Biosensor cells may display observable alterations in impedance, oxygen consumption, metabolic activity, or gene expression when exposed to saliva samples that include tumor-associated biomarkers, inflammatory mediators, or metabolic products from cancer cells. Integrated optical or electrical systems can detect these changes, enabling non-invasive screening procedures [85].

**Figure 6:**
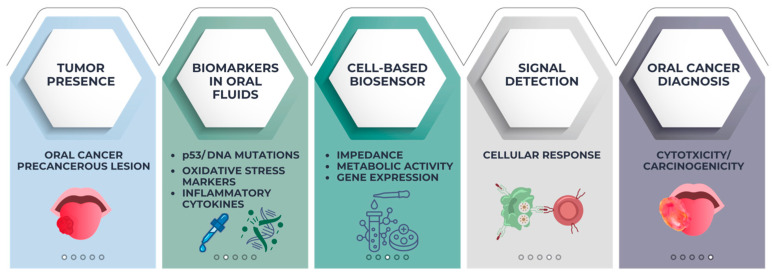
Principle of cell-based biosensors for oral cancer detection. Saliva and oral fluids may contain tumor-associated biomarkers such as DNA mutations, oxidative stress markers, inflammatory cytokines, and metabolic products. Cell-based biosensors detect cellular responses such as changes in impedance, metabolic activity, gene expression, or apoptosis, converting these biological effects into measurable electrical or optical signals for non-invasive oral cancer screening.

One important advantage of cell-based biosensors is their ability to detect biological impacts in addition to chemical presence. Thus, even in cases when the precise biomarker is not known, they are able to determine the sample’s potential cytotoxic or carcinogenic effects [86]. With so many biological pathways at play, this quality is crucial for cancer diagnosis. The principle of cell-based biosensors for oral cancer detection is illustrated in Figure 6.

#### 3.5.4. Cytotoxicity Testing of Dental Materials

Cytotoxicity testing is a crucial phase in the biological assessment of dental materials, given their direct and extended interaction with oral tissues, including gingiva, tooth pulp, mucosa, and bone [87]. The leaching of leftover monomers, breakdown products, metal ions, or other chemical entities from dental materials might provoke cellular toxicity, inflammation, allergic responses, or tissue damage [88]. Consequently, evaluating the cytotoxic potential of dental materials is crucial for confirming their biocompatibility and clinical safety.

Cell-based biosensors are widely used for evaluating the biocompatibility and cytotoxicity of dental materials, including composites, adhesives, cements, impression materials, and implant biomaterials used in regenerative dentistry [89]. Biocompatibility testing is essential because dental materials are in direct contact with oral tissues for long periods and may release monomers, metal ions, nanoparticles, or degradation products [90].

Chemical tests and cell cultures are the cornerstones of conventional cytotoxicity testing methods. These include 3-(4,5-dimethylthiazol-2-yl)-2,5-diphenyltetrazolium bromide (MTT), Lactate Dehydrogenase (LDH) release and apoptosis assays [91]. Nevertheless, biosensors based on cells enable the continuous tracking of cellular processes such as metabolism, adhesion, proliferation, and morphology without disrupting the cell culture. This is a characteristic application for methods like metabolic biosensors, optical biosensors, and electric cell–substrate impedance sensing (ECIS) [91].

The use of cell-based biosensors in cytotoxicity testing may improve the evaluation of dental material biocompatibility by providing dynamic information on cell behavior, toxicity thresholds, and long-term effects. This approach may also reduce the need for animal testing and improve the development of safer dental materials.

#### 3.5.5. Salivary Diagnostics and Biomarker Monitoring

Thanks to its non-invasive collection method, convenience of storage, and capacity to reflect both local oral problems and systemic health status, saliva has become an important diagnostic fluid in oral health research [92]. Detection, monitoring, and prognosis of oral disorders, such as dental caries, periodontal disease, oral cancer, and infections, are being improved by salivary diagnostics [93].

Salivary biomarkers include microbial components, enzymes, antibodies, nucleic acids (DNA and RNA), metabolites, and proteins, as well as non-specific indicators of inflammatory and oxidative stress responses, such as cytokines and reactive oxygen species [94]. When applied to the mouth, these biomarkers reveal important details about inflammation, tissue damage, microbial activity, and the immunological response. It should be noted that many salivary biomarkers, particularly cytokines and oxidative stress markers, are not disease-specific and may reflect general inflammatory or physiological conditions. In addition, their concentration levels can vary significantly depending on individual variability, disease stage, and sampling conditions.

From a biosensing perspective, analytical performance parameters such as sensitivity, specificity, and clinically relevant cut-off values are essential for accurate interpretation. However, these parameters are not consistently reported across studies, and standardized thresholds for many salivary biomarkers in oral diseases have yet to be established.

Saliva is great for real-time monitoring of oral illnesses since it moisturizes the mouth tissues directly [95]. Representative studies and the main salivary biomarkers used are summarized in Table 4.

In periodontal disease, several salivary biomarkers have been identified as indicators of inflammation and tissue breakdown. Among the most studied biomarkers are interleukins (IL-1β, IL-6, IL-8), tumor necrosis factor-alpha (TNF-α), matrix metalloproteinases, C-reactive protein and prostaglandin E2 [101]. Elevated levels of these biomarkers are associated with periodontal inflammation, connective tissue degradation, and alveolar bone resorption. Monitoring these biomarkers can help assess disease activity and treatment response [101].

The use of salivary diagnostics in the early diagnosis of oral cancer is very crucial. Researchers have found biomarkers in saliva from patients with oral squamous cell carcinoma, including p53 antibodies, Cyfra 21-1, lactate dehydrogenase (LDH), particular microRNAs, and DNA alterations [102]. These biomarkers have the potential to enhance prognosis by enabling earlier intervention in the event of malignant transformation.

The role of bacteria and demineralization in tooth caries is the primary focus of salivary diagnostics. Caries risk can be evaluated using biomarkers such as levels of Streptococcus mutans, lactobacilli, pH of saliva, buffering capacity, amounts of calcium, phosphate, and fluoride, and so on. Amylase, mucins, and proline-rich proteins are some of the salivary proteins that help preserve enamel and prevent microbe adherence [103].

Cell-based biosensors represent a promising approach for salivary diagnostics because they can detect biologically active molecules rather than only their presence. These biosensors use living cells engineered to respond to specific biomarkers such as inflammatory cytokines, bacterial toxins, or metabolic products [104]. When exposed to saliva containing these biomarkers, the cells generate measurable signals such as fluorescence, electrical changes, or metabolic activity variations. This allows rapid and sensitive detection of disease-related biomarkers [104].

Oral disorders can be monitored in real-time, pathological changes can be detected early, and personalized treatment plans can be developed with the help of cell-based biosensors combined with salivary diagnostics. The oral health condition of patients might soon be continuously monitored at dental offices or even at home with the help of portable salivary biosensor devices.

#### 3.5.6. Comparison of Cell-Based Biosensors with Emerging Liquid Biopsy Technologies

Recent advances in photonic and nanotechnology-based biosensors further highlight the rapid evolution of liquid biopsy platforms [105]. In recent years, liquid biopsy has emerged as a rapidly evolving field, encompassing a wide range of biosensing technologies for the detection of circulating and salivary biomarkers [106]. These include electrochemical, optical, plasmonic, nanophotonic, aptamer-based, and nucleic acid detection platforms, many of which have demonstrated high analytical sensitivity and specificity [107].

Compared to these approaches, cell-based biosensors represent a distinct strategy that relies on functional biological responses rather than direct molecular recognition. This enables the detection of complex cellular interactions and integrated biological effects, which may not be captured by conventional analytical systems [108].

However, cell-based biosensors may exhibit limitations in terms of specificity, standardization, and reproducibility, particularly when compared to highly optimized molecular detection platforms. In contrast, technologies such as nucleic acid-based assays and photonic sensors often provide lower limits of detection and higher analytical precision but may lack the ability to capture dynamic biological responses [109].

From a translational perspective, scalability, robustness, and clinical readiness remain key challenges across all platforms. While many molecular biosensors are closer to clinical implementation, cell-based systems offer unique opportunities for functional diagnostics and real-time monitoring of cellular behavior.

## 4. Challenges and Future Perspectives

Despite the potential of cell-based biosensors for the diagnosis and monitoring of oral diseases, numerous hurdles hinder their extensive clinical application. A primary constraint pertains to cellular stability and vitality. Given that these biosensors depend on living cells as sensing components, ensuring cell viability, repeatability, and consistent responses across time poses significant challenges. Alterations in cell culture conditions, temperature, nutrient accessibility, and storage parameters may affect sensor efficacy and result in unpredictability in outcomes.

A significant difficulty is the absence of standardization and validation. Numerous cell-based biosensor technologies remain in the experimental phase, necessitating extensive clinical validation investigations prior to their widespread application in dentistry practice. Clinical evaluations must assess sensitivity, specificity, repeatability, and long-term stability across varied patient groups.

The integration of microfluidic and lab-on-chip technologies presents both an opportunity and a challenge. Although microfluidic devices facilitate miniaturization, automation, and multiplex biomarker detection, challenges including device fabrication expenses, biofouling, calibration, and sample variability have to be resolved. Moreover, saliva is a multifaceted biological fluid affected by hydration, circadian rhythms, systemic diseases, drugs, and lifestyle variables, which may influence biomarker levels and sensor precision.

Regulatory approval and clinical implementation pose considerable challenges. Prior to the implementation of cell-based biosensors in conventional dentistry practice, it is essential to obtain regulatory approval, standardize devices, train clinicians, and integrate these technologies into dental workflows. Cost-effectiveness and user-friendliness will significantly influence their acceptance.

Despite the significant advances reported in the field of cell-based biosensors, the existing evidence base presents several limitations that should be acknowledged. A considerable proportion of studies are conducted under controlled in vitro conditions, which may not fully reflect the complexity of the oral environment and may lead to overestimation of analytical performance and clinical applicability.

In addition, many studies report promising sensitivity and specificity values without standardized validation protocols or direct comparison with established diagnostic methods, limiting the generalizability of the findings. The variability in cell models, experimental conditions, and detection platforms further complicates cross-study comparisons.

Moreover, potential conflicts of interest, including industry involvement in biosensor development and validation, are not always explicitly disclosed and may contribute to an over-optimistic interpretation of results. These factors highlight the need for standardized validation frameworks, independent replication, and clinically oriented studies to support the translation of cell-based biosensors into routine dental practice.

While numerous studies report promising results for cell-based biosensors, the evaluation of their practical applicability requires careful consideration of key analytical performance parameters. These include sensitivity, limit of detection, response time, reproducibility, stability, and selectivity, which are not consistently assessed or reported across the literature [110,111].

A significant proportion of published systems remain at the proof-of-concept stage, demonstrating feasibility under controlled laboratory conditions rather than validated performance in clinical settings. As a result, the reported analytical performance may not fully reflect real-world conditions [112].

In contrast, only a limited number of biosensor platforms, particularly those based on established molecular detection strategies, have progressed toward higher technology readiness levels, including clinical validation or commercialization [113].

Cell-based biosensors, while offering unique advantages in capturing functional biological responses, still face challenges related to standardization, robustness, and scalability. Therefore, a clearer distinction between experimental prototypes and clinically applicable systems is essential, and future research should prioritize validation under clinically relevant conditions to support translation into routine diagnostic practice.

Beyond analytical performance, the implementation of cell-based biosensors in practical diagnostic devices involves several system-level challenges. These include precise fluid handling within microfluidic environments, efficient cell encapsulation or immobilization, maintenance of long-term cell viability, and stable interfacing between living cells and transducer components. In addition, signal processing and data interpretation must account for biological variability and dynamic cellular responses.

A major limitation of cell-based systems is the inherent complexity associated with maintaining living cells in portable, miniaturized, or disposable platforms. Factors such as nutrient supply, waste removal, environmental control, and cellular stability significantly impact device performance and reproducibility.

In comparison, cell-free and synthetic biosensing platforms—such as nucleic acid-based assays or engineered molecular recognition systems—offer advantages in terms of stability, scalability, and ease of integration into point-of-care devices. However, these approaches may lack the ability to capture complex functional biological responses.

Although cell-based biosensors are frequently proposed as promising tools for real-time and chairside diagnostics, there remains a substantial gap between laboratory-scale prototypes and clinical implementation. Most reported systems are evaluated under controlled experimental conditions, which do not fully capture the complexity and variability of real biological samples [114].

In particular, saliva as a diagnostic medium presents significant challenges, including variability in composition, flow rate, pH, and the presence of interfering substances, all of which may affect sensor performance and reproducibility. In addition, inter-patient variability further complicates the establishment of reliable diagnostic thresholds [115].

From a translational perspective, regulatory approval, lack of standardized protocols, and the need for robust validation under clinically relevant conditions represent major barriers. Current systems often lack the robustness and reproducibility required for routine clinical use [116].

In addition to cell-based biosensors, several alternative biosensing paradigms have gained increasing attention in recent years, including cell-free systems, synthetic gene circuits, and organ-on-chip platforms. These approaches offer important advantages, particularly in terms of stability, reproducibility, and ease of integration into portable diagnostic devices.

Cell-free biosensors, for example, eliminate the need for maintaining living cells and are therefore more robust and easier to standardize. Similarly, synthetic gene circuits enable programmable sensing with high specificity, while organ-on-chip systems provide more physiologically relevant models by mimicking tissue-level organization and function.

However, these approaches may not fully capture the dynamic and integrated responses of living cells to complex biological stimuli. In this context, cell-based biosensors offer a unique advantage by enabling functional readouts of cellular behavior, including responses to multiple interacting factors.

Recent advances in biosensing technologies have been strongly driven by the development of nanophotonic and integrated photonic platforms, particularly in the context of liquid biopsy diagnostics. These systems offer high analytical sensitivity, rapid response times, and the potential for real-time detection of circulating biomarkers in complex biological fluids [106].

Nanophotonic biosensors, including plasmonic, fluorescence-based, and Raman-enhanced systems, benefit from enhanced light–matter interactions at the nanoscale, enabling improved signal amplification and reduced detection limits [117].

Compared to cell-based biosensors, these technologies often provide superior analytical precision and lower limits of detection, in some cases reaching femtomolar or even lower concentration ranges. However, they typically rely on direct molecular recognition and may not capture the complex functional responses of living cells [118].

A key limitation of the current literature is the lack of standardized reporting of quantitative performance metrics, which limits direct comparison between biosensor platforms.

Notwithstanding these obstacles, the prospects for cell-based biosensors in oral health are exceedingly optimistic. Progress in microfluidics, organ-on-chip systems, synthetic biology, and nanotechnology is anticipated to enhance the sensitivity, stability, and portability of biosensors.

Portable and chairside biosensor devices provide swift, non-invasive diagnosis, real-time illness monitoring, and tailored therapy planning. In the future, salivary biosensors combined with digital health platforms and artificial intelligence may provide continuous monitoring of dental and systemic health, hence advancing precision dentistry and personalized medicine.

## 5. Conclusions

Cell-based biosensors represent an emerging and highly promising technology for the detection, monitoring, and understanding of oral diseases. Unlike conventional diagnostic methods that detect only the presence of microorganisms or biomarkers, cell-based biosensors are capable of detecting biologically active molecules and evaluating cellular responses such as inflammation, cytotoxicity, oxidative stress, and metabolic changes. This functional approach provides more clinically relevant information regarding disease activity, progression, and treatment response.

Cell-based biosensors have various uses in oral health, including the detection of periodontal disease, monitoring of cariogenic biofilm activity, early identification of oral cancer, cytotoxicity assessment of dental materials, and salivary diagnostics. These devices possess the capability to enhance early diagnosis, risk evaluation, and individualized treatment planning, hence reinforcing the principle of precision dentistry.

The integration of cell-based biosensors with salivary diagnostics, microfluidic systems, and lab-on-chip technologies represents a major step toward the development of portable, chairside, and real-time diagnostic devices. Such technologies could allow continuous monitoring of oral health, early detection of pathological changes, and rapid evaluation of treatment effectiveness.

Nonetheless, several obstacles continue to impede the extensive clinical application of cell-based biosensors, encompassing concerns regarding cell stability, standardization, repeatability, cost, clinical validation, and regulatory licensing. Additional research is necessary to enhance biosensor stability, establish standardized techniques, and validate these technologies in extensive clinical trials.

Despite these limitations, advances in microfluidics, organ-on-chip technologies, synthetic biology, and nanotechnology are expected to significantly improve the performance and clinical applicability of cell-based biosensors. In the future, these technologies may become important tools in oral diagnostics, enabling non-invasive, rapid, and personalized monitoring of oral diseases and contributing to the development of precision and digital dentistry.

## Figures and Tables

**Table 1:** Comparative overview of cell-based biosensor technologies used in oral health.

Biosensor Type	Cell Models	Target Biomarkers	Detection Principle	Advantages	Limitations
Electrochemical	HGFs, THP-1	Cytokines (IL-6, TNF-α)	Impedance/current changes	High sensitivity	Signal variability
Optical	SCC-9, epithelial cells	Cancer markers	Fluorescence	Non-invasive	Lower stability
Impedance-based	Fibroblasts, immune cells	Inflammatory markers	Cell impedance	Real-time monitoring	Complex setup
Microfluidic	Mixed cell systems	Salivary biomarkers	Lab-on-chip	Rapid detection	Integration challenges

**Table 2:** Challenges and limitations of microfluidic and lab-on-chip technologies in periodontal diagnostics.

Challenge	Description	Impact on Clinical Use
Device standardization [59]	Lack of standardized protocols and device designs across different microfluidic platforms	Difficult comparison between studies and limited clinical implementation
Reproducibility [60]	Variability in chip fabrication, sensor sensitivity, and biological components	Reduced reliability and consistency of diagnostic results
Calibration [61]	Sensors require precise calibration for biomarker detection	Incorrect calibration may lead to inaccurate biomarker measurements
Sample variability [62]	Saliva composition varies depending on hydration, circadian rhythm, medication, smoking, and systemic diseases	May influence biomarker concentration and diagnostic accuracy
Biofouling [63]	Accumulation of proteins, cells, and debris on microfluidic surfaces	Reduced sensor performance and signal interference
Cost-effectiveness [64]	High development and fabrication costs of microfluidic devices	Limits widespread use in routine dental practice
Clinical validation [65]	Limited large-scale clinical studies validating these technologies	Delays regulatory approval and clinical adoption
Regulatory approval [66]	Medical device approval processes are complex and time-consuming	Slows translation from research to clinical practice
Integration into dental workflow [67]	Need for user-friendly devices suitable for chairside use	Requires adaptation of clinical workflow and training

**Table 3:** Cell types, biomarkers, detection methods, and clinical applications of cell-based biosensors in periodontal disease research.

Target	Cell Type Used	Biomarkers Detected	Detection Method	Clinical Application
Periodontal pathogens [74]	Gingival epithelial cells	Cytokines, ROS	Optical/Electrochemical	Detection of periodontal infection
Inflammatory response [75]	Fibroblasts	IL-1β, IL-6, TNF-α	Impedance	Monitoring inflammation
Tissue destruction [76]	Osteoblast-like cells	MMP-8, MMP-9	Electrochemical	Monitoring tissue breakdown
Host response [77]	Immune cells	Cytokines, oxidative stress markers	Optical/Impedance	Personalized periodontal diagnostics

**Table 4:** Salivary biomarkers associated with oral diseases and their diagnostic applications.

Study	Oral Disease	Salivary Biomarkers	Detection Method	Main Findings
Lee et al., 2009 [96]	Oral cancer	IL-8, IL-1β	ELISA	Elevated cytokines associated with oral cancer
Ramseier et al., 2009 [97]	Periodontitis	MMP-8, IL-1β	Immunoassay	Biomarkers correlated with periodontal inflammation
Zhang et al., 2010 [98]	Oral cancer	Salivary RNA biomarkers	PCR	Salivary RNA useful for oral cancer detection
Kaufman & Lamster, 2002 [99]	Oral diseases	Multiple salivary biomarkers	Biochemical assays	Saliva useful diagnostic fluid
Javaid et al., 2016 [100]	Caries/Periodontitis	Microbial and inflammatory markers	Review	Salivary biomarkers useful for oral disease monitoring

## Data Availability

No new data were created or analyzed in this study. Data sharing is not applicable to this article.

## Abbreviations

The following abbreviations are used in this manuscript:

GFP	Green Fluorescent Protein
MTT	3-(4,5-dimethylthiazol-2-yl)-2,5-diphenyltetrazolium bromide
LDH	Lactate Dehydrogenase
TNF-α	Tumor Necrosis Factor-Alpha
ECIS	Electric Cell–Substrate Impedance Sensing
